# Macrophage phagocytosis of human norovirus-infected cells in an *ex vivo* human enteroid-macrophage coculture model

**DOI:** 10.1128/mbio.01180-25

**Published:** 2025-07-09

**Authors:** Ngan Fung Li, Sue E. Crawford, Sydney R. Mittiga, Cristian Coarfa, Hoa Nguyen-Phuc, Budi Utama, Sarah E. Blutt, Sasirekha Ramani, Mary K. Estes

**Affiliations:** 1 Department of Molecular Virology and Microbiology, Baylor College of Medicine 3989, Houston, Texas, USA; 2 Dan L. Duncan Cancer Center, Baylor College of Medicine 3989, Houston, Texas, USA; 3 Department of Molecular and Cellular Biology, Baylor College of Medicine, Houston, Texas, USA; 4 Center for Precision and Environmental Health, Baylor College of Medicine 3989, Houston, Texas, USA; 5 Shared Equipment Authority, Rice University, Houston, Texas, USA; 6 Texas Medical Center Digestive Disease Center Gastrointestinal Experimental Model Systems (GEMS) Core, Houston, Texas, USA; 7 Department of Medicine, Baylor College of Medicine 3989, Houston, Texas, USA; Duke University School of Medicine, Durham, North Carolina, USA

**Keywords:** human norovirus, intestinal enteroids, macrophages, phagocytosis, innate immunity

## Abstract

Human norovirus (HuNoV) causes acute gastroenteritis in immunocompetent hosts and chronic infection in immunocompromised individuals. Many recent studies of replication and innate immune responses following HuNoV infection have utilized epithelium-only human intestinal enteroids (HIEs), which lack immune cells. Here, we utilized an *ex vivo* enteroid-macrophage coculture model consisting of HIEs and different subtypes of human peripheral blood mononuclear cell-derived macrophages to better recapitulate *in vivo* gut biology and explore the role of macrophages in HuNoV replication and pathogenesis. We show that HuNoV infection in HIEs polarized on Transwells leads to bilateral release of the viral genome, with predominant apical virus release. Coculture with naïve M0, pro-inflammatory M1, or anti-inflammatory M2 macrophages does not change levels of HuNoV replication. We found that macrophages respond to HuNoV infection by phagocytosis of virus-infected cells with pro-inflammatory M1 macrophages exhibiting the greatest phagocytic activity. Apical release of chemokines (IP-10, MIP-1α, and RANTES), acute-phase inflammatory mediators (IL-1α, IL1-RA, IL-6, and TNF-α), and anti-inflammatory mediators IL-10 and IL-1RA are increased following HuNoV infection only in HIEs cocultured with activated macrophages. These findings underscore the importance of epithelial-macrophage crosstalk in chemokine production during the early stages of infection required for leukocyte recruitment and initiation of the host response.

**IMPORTANCE:**

Human noroviruses (HuNoVs) are important human enteric pathogens that cause outbreaks and sporadic gastroenteritis in people of all ages. HuNoV-induced illness can become chronic and debilitating in immunocompromised hosts. Previously, we observed an uptake of HuNoV-infected epithelial cells by macrophages in intestinal biopsies derived from chronically infected transplant patients. Based on this, we put forward the hypothesis that intestinal macrophages are important in contributing to an anti-HuNoV response during the early stages of infection. Here, we investigated the potential role of different macrophage subtypes in the induction of immune responses following HuNoV infection using HIE-macrophage coculture models. We found that pro-inflammatory macrophages showed the greatest capacity for phagocytosing virus-infected cells. This study highlights the importance of the interaction of the intestinal epithelium with activated macrophages in an anti-viral response that may be critical for enhancing viral clearance and reducing viral spread.

## INTRODUCTION

Human norovirus (HuNoV) belongs to the *Caliciviridae* family of single-stranded positive sense RNA viruses that causes viral gastroenteritis worldwide. Transmission is achieved via the fecal-oral route, contaminated food, water, and environmental surfaces or by direct contact or aerosols during vomiting. HuNoV illness is typically self-limiting in immunocompetent people, characterized by stomach pain, nausea, vomiting, and diarrhea within 12–48 h of exposure (1–3). Despite the high burden of disease, there is no specific therapy for treating HuNoV infection, and interventions focus on alleviating symptoms including electrolyte and fluid replacement.

Efforts to understand the induction of HuNoV-specific innate immune responses in people primarily come from controlled human infection models and observational reports from naturally acquired infection (4–9). A study in travelers showed an acute T helper(Th) 1 mucosal response characterized by fecal detection of interleukin (IL)-2, IL-6, and interferon(IFN)-γ during the acute phase of HuNoV-induced gastroenteritis (8). Serum cytokine analyses of healthy volunteers infected with the prototype GI.1 HuNoV revealed a mixed Th1/Th2 response, with elevation in pro- and anti-inflammatory cytokines including IL-2, IL-4, IL-6, IL-10, tumor necrosis factor-alpha (TNF-α), and IFN-γ as well as chemokines associated with monocytes, neutrophils, and leukocyte recruitment (5, 6). This observation was confirmed in a case report within a clinical trial where an increase in mobilization and activation of monocytes, neutrophils, natural killer cells, and regulatory T cells was reported (4). Together, these data suggest a coordinated interplay between the intestinal epithelium and innate and adaptive immunity in controlling HuNoV infection. What remains elusive are details about the cellular and molecular mechanisms that occur at the mucosal infection sites.

Progress in delineating the cellular mechanisms of HuNoV infection and innate immunity at the mucosa was previously hindered by the lack of a robust *in vitro* culture system that permits HuNoV replication. This was overcome by the recent use of tissue-derived human intestinal enteroids (HIEs). These non-transformed cultures have successfully enabled the replication of multiple HuNoV strains and identification of host factors critical for HuNoV replication (10, 11). HuNoV replication occurs in enterocytes and potentially enteroendocrine cells (10, 12, 13). A histological comparison of tissue biopsies in infected and uninfected (control) immunocompromised transplant patients showed the presence of the HuNoV major viral capsid antigen (VP1) and the non-structural antigens (RNA-dependent RNA polymerase and VPg) only in enterocytes indicative of replicating virus in infected individuals (14). More importantly, in these biopsies, CD68-positive macrophages in the lamina propria colocalized with VP1 and cytokeratin-8 (CK8), a marker for epithelial cells, suggesting that macrophages engulf virus-infected epithelial cells. These findings shed light on macrophages as potential innate immune responders during HuNoV infection. While our previous work demonstrated that monocyte-derived macrophages do not support HuNoV replication (15), only limited data exist on HuNoV replication and pathogenesis within a multicellular *in vitro* coculture system that recapitulates the complex cellular interactions in the human intestinal mucosa (16). The research reported herein aimed to address this knowledge gap by exploring the role of macrophages in an acute HuNoV replication and the epithelial-macrophage response using a coculture system comprising of human small intestine-derived epithelial cells and macrophages.

## RESULTS

### Coculture with different macrophage subtypes does not induce changes in barrier function of differentiated HIEs

**Fig 1:**
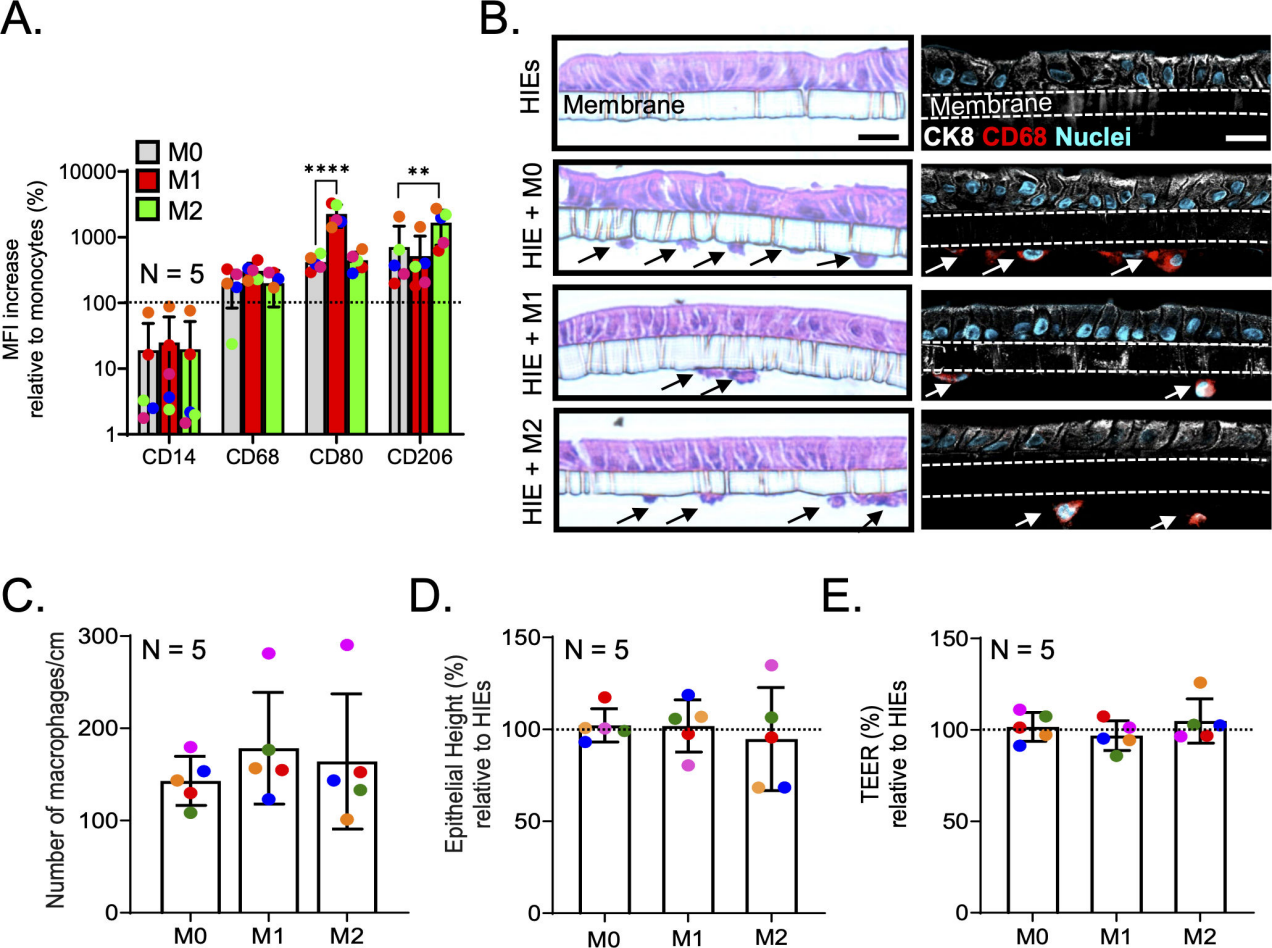
HIEs cocultured with all macrophages subtypes remain structurally and morphologically intact. (A) Expression of CD14, CD68, CD80, and CD206 in macrophages relative to monocytes derived from five donors. Dotted line indicates 100% in mean fluorescence intensity (MFI). Gray, red, and green bars indicate M0, M1, and M2 macrophages, respectively. Data are presented as mean ± SD. Statistical significance was determined using Dunnett’s multiple comparisons test, comparing the relative expression between macrophage phenotypes. **P < 0.01, ****P < 0.0001. The gating stratgey and superimposed plots displaying macrophage heterogeneity from individual donors are shown in Fig. S1 and Fig. S2. (**B**) H&E and immunofluorescence images showing adherent M0, M1, and M2 macrophages (CD68; red) to differentiated HIEs (CK8; white) at 1 day post coculture indicated by arrows. Scale bar = 20 µm. (**C**) Number of adherent macrophages per centimeter of HIE monolayer. Measurement of (**D**) epithelial cell height and (**E**) barrier integrity in HIE-macrophage cocultures relative to HIEs alone. Data are presented as mean ± SD and compiled from five donors. Each color corresponds to macrophages derived from the PBMCs of a single donor. Area-normalized TEER values for pre- and post-coculture conditions are shown in Fig. S3 .

Our previous study in patient biopsies indicated that macrophages engulf HuNoV-infected epithelial cells (14). To evaluate if a specific macrophage subtype is responsible for epithelial engulfment, we established macrophage-HIE cocultures using human jejunal enteroids grown on Transwells and either naïve (M0), pro-inflammatory (M1), or anti-inflammatory (M2) macrophages. The generation of respective macrophage subtypes was confirmed by classical surface markers for naïve and activated macrophages: CD14 (monocyte/macrophage), CD68 (pan-macrophage), CD80 (pro-inflammatory M1 macrophage), and CD206 (anti-inflammatory M2 macrophages) (17, 18). Our results showed an increase in CD68 expression for all macrophage subtypes, with a significant increase in CD80 expression in M1 macrophages (*P* < 0.0001) and CD206 expression (*P* < 0.01) in M2 macrophages compared to M0 macrophages (Fig. 1A). For establishing an HIE-macrophage coculture system optimal for HuNoV replication, we selected a commercial medium, IntestiCult Organoid Growth Medium (OGM) because we showed enhanced replication of several HuNoV strains in HIEs cultured with OGM (19, 20). The presence of adherent macrophages was confirmed by hematoxylin and eosin (H&E) and immunofluorescence labeling by CK8 and CD68 for epithelial cells and macrophages, respectively (Fig. 1B), with a consistent number of adherent macrophages observed in all three coculture systems (Fig. 1C). Epithelial height and transepithelial resistance (TEER) measurements were performed as previously reported, comparing results before and 1 day after HIEs were cocultured with macrophage subtypes (21–24). Integration of M0 and M1 macrophages to HIEs did not cause a significant change in epithelial cell height (Fig. 1D). Similarly, there was no change in epithelial cell height in HIEs cocultured with M2 macrophages although more variability was observed with cells from two donors showing a reduction in epithelial cell height (with an average of 68% ± 0.1%) and one donor showing a relative increase of 134.8%. No change in barrier integrity, measured by TEER, was observed in HIEs cocultured with both naive and activated macrophages compared to HIEs alone (Fig. 1E). Our data show a successful integration of either naive or activated macrophages to HIEs differentiated in OGM medium, and the presence of macrophages does not alter the epithelial cell height nor the barrier integrity.

A previous study reported an increase in barrier function in differentiated HIE monolayer in the presence of M0 macrophages (25). Since different media were used between our study and the previous publication, we conducted a side-by-side comparison to assess how HIEs cultured in different culture media (Wnt3A-free differentiation medium vs OGM medium) respond to the presence of macrophages (Fig. S4). Our results showed that HIEs differentiated in Wnt3A-free compared to OGM media displayed divergent epithelial responses to macrophage integration. In HIEs differentiated using Wnt3A-free medium, we observed an apparent increase in barrier integrity when cocultured with M0 and M2 macrophages (Fig. S4A). A significant reduction in barrier integrity was observed in HIEs cocultured with M1 macrophages (*P* < 0.01). Interestingly, integration of M0, M1, and M2 macrophages with differentiated HIEs cultured in OGM medium did not alter barrier integrity (Fig. S4B). Our data are in agreement with Noel et al. (25) that the addition of M0 macrophages appears to enhance barrier integrity only in differentiated HIEs cultured with Wnt3A-free differentiation medium but not with OGM medium.

### Replication of HuNoV is not altered by integration of macrophages with differentiated HIEs

**Fig 2:**
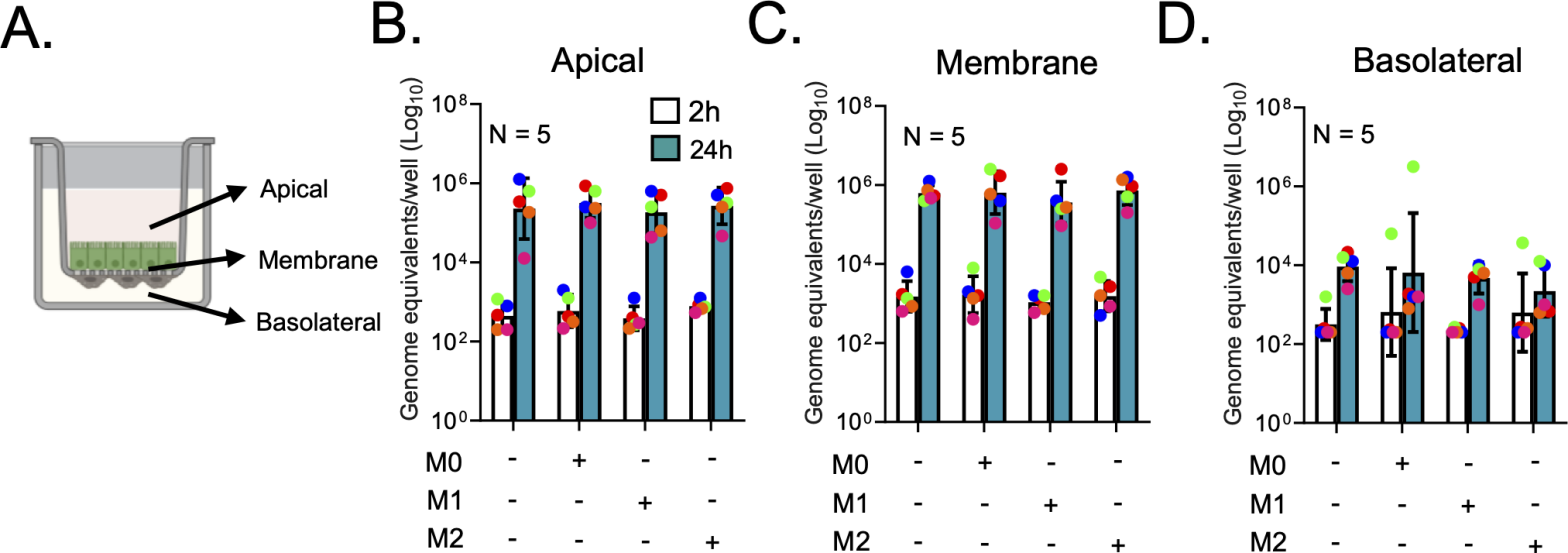
HuNoV replication is not altered in the presence of macrophages. (A) A schematic diagram showing the compartmentalization (apical, membrane, and basolateral) of HIE-macrophage cocultures in Transwells. HIEs and HIEs cocultured with M0, M1, or M2 macrophages were inoculated with HuNoV at 2.5 × 10^5^ genome equivalents per well. Virus replication was evaluated by assessing viral RNA levels in (**B**) apical, (**C**) membrane, and (**D**) basolateral compartments at 2 h and 24 h post infection. Data are presented as means ± SD. Each color corresponds to monocyte-derived macrophages derived from the PBMCs of a single donor used for one coculture experiment. Macrophages derived from five donors were tested.

To test whether HuNoV replication in the HIEs is enhanced by the presence of naive or activated macrophages, we performed HuNoV infection using HuNoV-positive stool filtrates in OGM-differentiated HIEs and HIE-macrophage cocultures and quantified genome equivalents (GE) using RT-qPCR across three coculture compartments: apical, membrane, and basolateral fractions at 2 and 24 hpi (Fig. 2A). In addition to a medium-treated group, we included γ-irradiated HuNoV-positive stool as another control to account for potential factors in stool inoculum that may influence HuNoV replication in the context of different macrophage subtypes. Our data demonstrated a bilateral release of viral genome in HIEs infected with HuNoV, where we observed a 2 log_10_ increase in GE in the apical compartment (Fig. 2B) and a 1.6 log_10_ increase in GE in the basolateral compartment (Fig. 2D). A 1.8 log_10_ increase in GE was observed in the membrane fractions of infected HIEs (Fig. 2C). The presence of all macrophage subtypes did not alter the increase in GE in either the apical, membrane, or basolateral compartments (Fig. 2B through D). TEER measurements indicated that neither HuNoV infection nor γ-irradiated virus compromised the barrier integrity of HIEs at 24 hpi (Fig. S5). Our data showed that newly synthesized viral genome is released bilaterally with a predominant apical release. This change in viral genome was not influenced by the presence of M0, M1, or M2 macrophages.

### Phagocytosis by macrophages was observed for HuNoV-infected epithelial cells in HuNoV-infected HIE-macrophage cocultures

**Fig 3:**
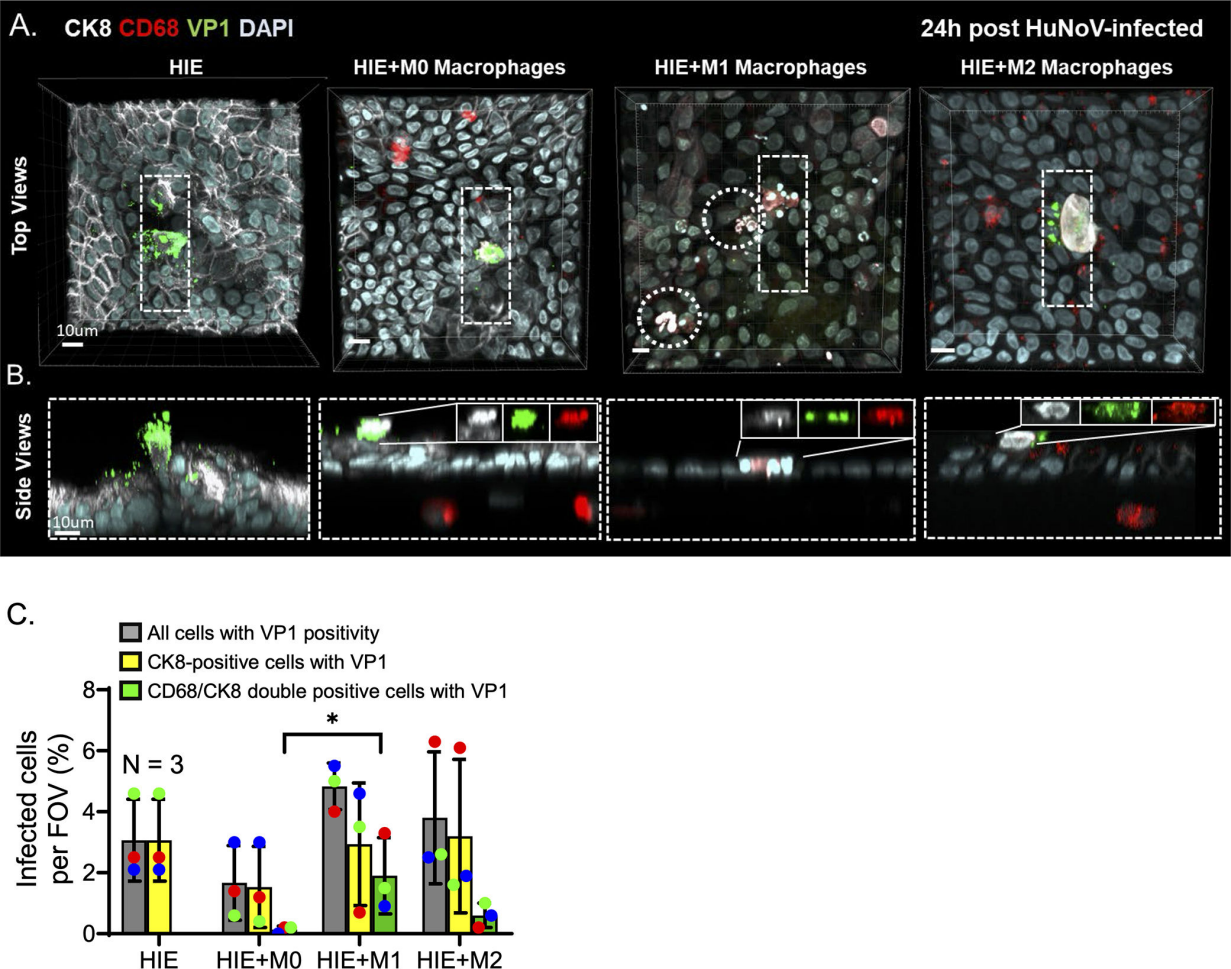
Phagocytosis of HuNoV-infected epithelial cells by all macrophage subtypes. Immunofluorescence labelling of HuNoV structural antigen (VP1; green) in epithelial cells (CK8; white) and macrophages (CD68; red) with (**A**) top views and (**B**) orthogonal views. Regions containing CK8/VP1/CD68-positive cells are highlighted with dotted outlines, and orthogonal views are illustrated from the boxed areas. (**C**) Percent of HuNoV-infected cells in infected HIEs and HIE-macrophage cocultures at 24 hpi. Data are represented as mean ± SD and compiled from three experiments. Each color corresponds to monocyte-derived macrophages derived from the PBMCs of a single donor used for one coculture experiment. Statistical significance in the number of CD68/CK8 double-positive cells with VP1 positivity was assessed in infected HIEs cocultured with either M0 or M1 macrophages using Dunnett’s multiple comparisons test; *P < 0.05.

To investigate whether macrophages phagocytose HuNoV-infected epithelial cells, fluorescence microscopy was used to detect the location of HuNoV major capsid antigen (VP1) expression in the infected HIE monolayers at 24 hpi. Colocalization of CK8 and VP1 was observed in infected HIEs with or without macrophages (Fig. 3A). Several VP1-positive cells were positive for both CK8 and CD68 within the epithelial layer in HuNoV-infected HIE-macrophage cocultures (Fig. 3B). Quantification of VP1 revealed an average HuNoV infection of 3.0% ± 1.1% per FOV in infected HIEs, compared to 1.7% ± 1.0%, 4.8% ± 0.6%, and 3.8% ± 1.8% in HIEs cocultured with M0, M1, and M2 macrophages, respectively (Fig. 3C). VP1/CK8-positive cells associated with CD68-positive cells were lower in HIEs cocultured with M0 (0.1% ± 0.1%) and M2 (0.6% ± 0.3%) macrophages but significantly higher with M1 macrophages (1.9% ± 1.0%, *P* < 0.05 compared to M0 macrophages) (Fig. 3C). VP1 positivity was only detected in CD68-positive cells that migrated to, not below the HIE monolayer. No VP1 positivity or macrophage phagocytosis was observed in mock-treated and γ-irradiated HuNoV-infected HIEs cocultured with macrophage. In summary, our data showed the presence of HuNoV viral capsid antigen VP1 in CD68/CK8 double positive cells localized to the apical compartments of HIEs cocultured with all macrophage subtypes. This association was more pronounced in HIEs cocultured with M1 macrophages compared to HIEs cocultured with M0 and M2 macrophages. In line with our previous report (14), our study confirms that macrophages recognize and phagocytose HuNoV-infected cells. Overall, we identified that pro-inflammatory M1 macrophages exhibited the greatest phagocytosis of infected epithelial cells compared to other macrophage subtypes.

### Induction of cytokines following HuNoV infection in HIEs cocultured with activated macrophages

**Fig 4:**
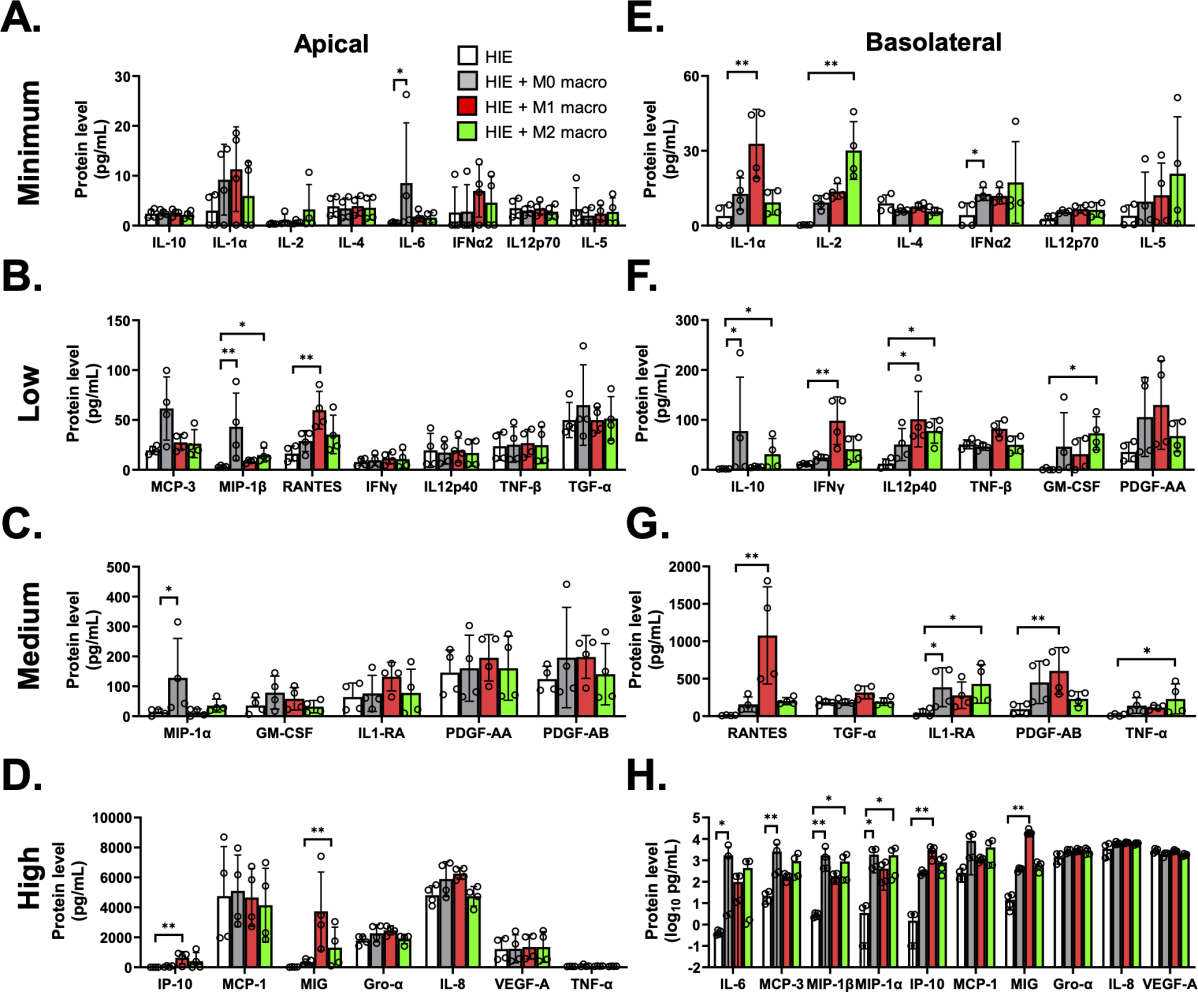
The presence of both naïve and activated macrophages induces bilateral secretion of cytokines in differentiated HIEs. Levels of 27 cytokines measured in apical (**A, B, C, D**) and basolateral (**E, F, G, H**) compartments of HIEs (white) and HIEs cocultured with M0 (gray), M1 (red), and M2 (green) macrophages. Cytokines are grouped into minimum, low, medium, and high panels based on similar detected levels for better visualization of data. Data are represented as mean ± SD and compiled from four donors. Each dot represents PBMC-derived macrophages used in HIE-macrophage coculture. Statistical significance was determined using a one-way ANOVA with Kruskal-Wallis test; *P < 0.05 and **P < 0.01.

To assess the cytokine response following HuNoV infection, we first characterized the baseline response in HIEs exposed to different macrophage subtypes (Fig. 4). Apical levels of IL-6 (*P* < 0.05), MIP-1α (*P* < 0.05), and MIP-1β (*P* < 0.01) were significantly higher in HIE cocultured with M0 macrophages compared to HIEs alone. Significantly higher levels of RANTES (*P* < 0.01) and IP-10 (*P* < 0.01) were observed in HIE cocultured with M1 macrophages and MIG (*P* < 0.01) and MIP-1β (*P* < 0.05) levels in HIEs cocultured with M2 macrophages. The profile of cytokines secreted also depended on the types of macrophage in the coculture. Basolateral levels of IFN-α2 (*P* < 0.05), IL-10 (*P* < 0.05), IL1-RA (*P* < 0.05), IL-6 (*P* < 0.05), MCP-3 (*P* < 0.01), MIP-1α (*P* < 0.05), and MIP-1β (*P* < 0.01) were significantly higher in HIEs cocultured with M0 macrophages. M1 macrophages triggered significant increases in IL-1α (*P* < 0.01), IFN-γ (*P* < 0.01), IL12p40 (*P* < 0.05), RANTES (*P* < 0.01), PDGF-AB (*P* < 0.01), IP-10 (*P* < 0.01), and MIG (*P* < 0.01). M2 macrophages significantly elevated levels of IL-2 (*P* < 0.01), IL-10 (*P* < 0.05), IL12p40 (*P* < 0.05), GM-CSF (*P* < 0.05), IL1-RA (*P* < 0.05), TNF-α (*P* < 0.05), MIP-1α (*P* < 0.05), and MIP-1β (*P* < 0.05). Fold increases in cytokine secretion in HIE-macrophage cocultures normalized to HIEs alone from individual donors, and pooled data from all donors are also shown in Fig. S6 and S7. Cytokine response also depends on the type of macrophages in the coculture models. Using a sparse partial least squares discriminant analysis (sPLSDA) approach, we showed group separation by cytokine response between HIE-macrophage cocultures compared to HIEs alone (Fig. S8). While this was less defined based on apically secreted cytokine response (Fig. S8A), a distinct separation was observed for cytokine responses in the basolateral compartments (Fig. S8B). The basolateral cytokine response in HIEs cocultured with M0 and M2 macrophages was comparable, which was consistent with findings by Hickman et al. showing M0 and M2 macrophages exhibiting similar secretory profiles compared to M1 macrophages (26). IP-10, MIG, and IFN-γ were the top three cytokines driving group separation in HIE-M1 macrophage cocultures (Fig. S8C).

**Fig 5:**
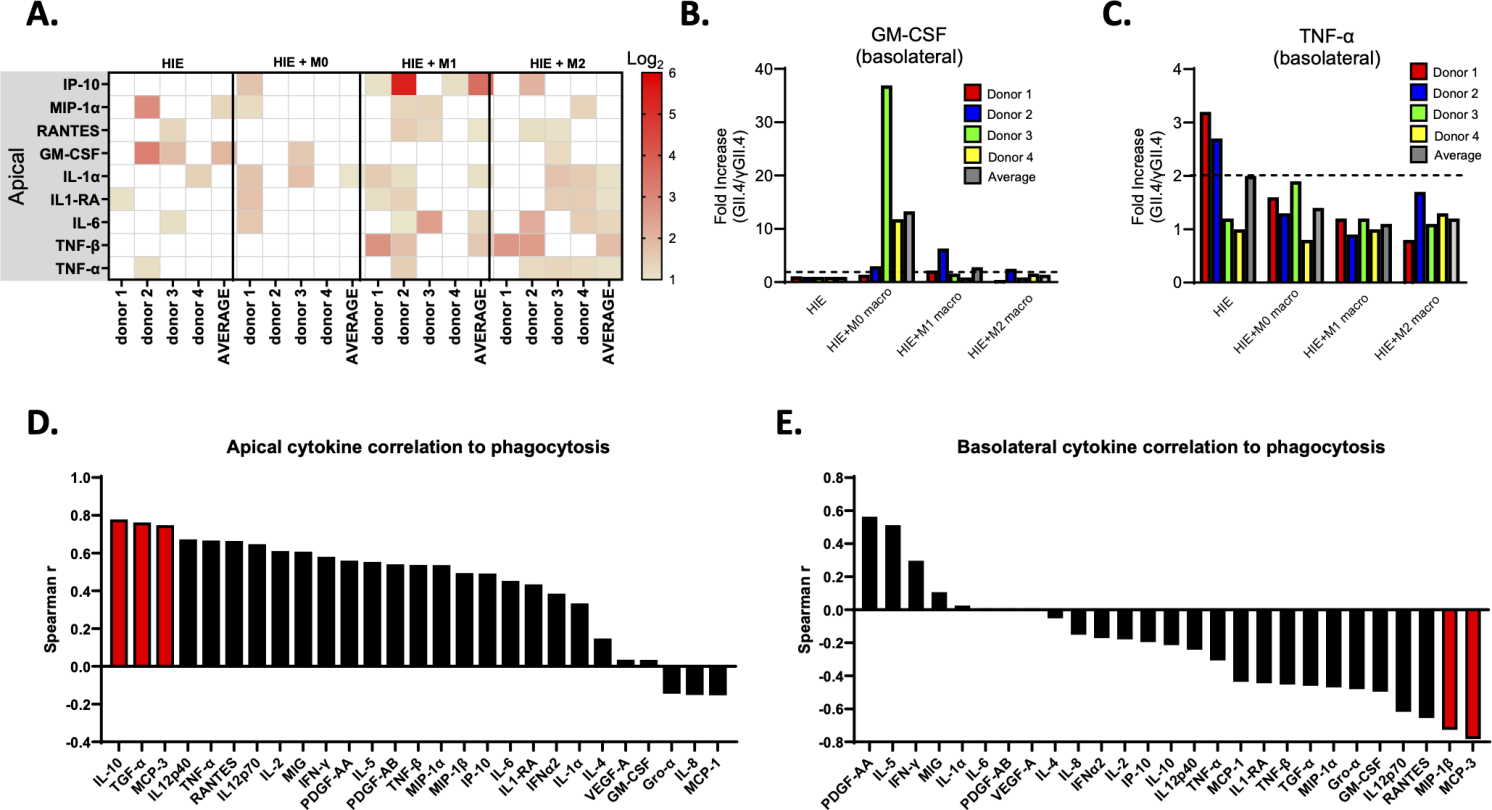
Chemokines and acute-phase inflammatory mediators were produced apically in HuNoV-infected HIEs cocultured with activated macrophages. Supernatants collected apically and basolaterally from the 24h post HuNoV-infected HIEs and HIE-macrophage cocultures were assessed for cytokine secretion. Twofold increases in the cytokine levels of the HuNoV-infected group normalized to the γ–irradiated HuNoV-infected group are shown. (**A**) A heatmap showing the log_2_ fold increases in cytokine levels. The *x*-axis displays the number of PBMC donors used for HIE-macrophage cocultures and the average value across all donors. Cells with less than a twofold change are depicted as white. Fold increases in the levels of (**B**) GM-CSF and (**C**) TNF-α secreted basolaterally. Dashed line indicates a twofold change. Waterfall plots of Spearman correlation analysis for fold increase in cytokine secreted (D) apically and (**E**) basolaterally to the phagocytosis index, defined by the number of CD68/CK8 double positive cells with VP1 positivity in HIE-macrophage coculture at 24 hpi. This correlation analysis was performed for donors 2, 3, and 4. Cytokines with statistically significant correlation to phagocytosis are labeled in red bars. Spearman *r* > 0.75, *P* < 0.05

Next, we assessed cytokine responses following HuNoV infection. A twofold increase in cytokine levels following HuNoV infection normalized to the γ-irradiated HuNoV control group was set as the threshold for infection-induced responses (Fig. 5A through C). While donor-to-donor differences were observed in cytokine responses with the different coculture models, overall, we observed an increase in several chemokines and acute-phase inflammatory mediators secreted apically in at least two out of the four donors in the infected HIEs cocultured with M1 and M2 macrophages (Fig. 5A). This includes chemokines involved in leukocyte recruitment such as IP-10, MIP-1α, and RANTES as well as the acute-phase inflammatory cytokines IL-1α, IL1-RA, IL-6, TNF-α, and the adaptive immune mediator TNF-β. An increase in apically secreted GM-CSF was observed in two out of four donors in infected HIEs, but not in infected HIEs cocultured with macrophages. HuNoV-induced basolateral cytokine secretion was minimal, whereby a twofold increase in the expression of GM-CSF (Fig. 5B) and TNF-α (Fig. 5C) in infected HIEs cocultured with M0 macrophages and HIEs alone was observed in two out of four donors, respectively.

In addition to characterizing the cytokine responses, we also assessed the relationship between fold increases in cytokines following HuNoV infection to the observed phagocytosis by macrophages (Fig. 3). We conducted a correlation analysis to examine the relationship between the phagocytosis index and cytokines secreted apically (Fig. 5D) and basolaterally (Fig. 5E) in donors 2, 3, and 4. A significant positive correlation (Spearman *r* > 0.75, *P* < 0.05) was observed between the phagocytosis index and apical levels of IL-10, TGF-α, and MCP-3, while a significant negative correlation (Spearman *r* < −0.7, *P* < 0.05) was observed with levels of MIP-1β and MCP-3 secreted basolaterally.

## DISCUSSION

Knowledge of HuNoV-mediated innate immunity is continuously evolving, accelerated by the use of human-derived intestinal enteroids (HIEs) as a platform for studying the basic biology of HuNoV. Several reports indicate that macrophages in the lamina propria may contribute to the host responses toward HuNoV (12, 14). We addressed this by utilizing an established enteroid-macrophage coculture model previously used to study bacterial infections and explored the role of both naïve and activated macrophages during acute HuNoV replication (25, 27). First, we showed a bilateral but predominately apical release of viral genome in differentiated HIEs polarized on Transwells infected with HuNoV. At this timepoint, no barrier dysfunction was detected. Second, the addition of macrophage subtypes with differentiated HIEs did not affect HuNoV infection or replication, consistent with the hypothesis that the intestinal epithelium serves as the primary site of HuNoV infection. Unlike murine noroviruses, which are known to target immune cells for primary infection (28), the role of intestinal macrophages as a permissive cell type for HuNoV replication or solely in phagocytosis remains a subject of debate. Attempts to elucidate the permissiveness of macrophages to HuNoV replication have been explored previously by two studies. We previously reported that human monocyte-derived naïve macrophages do not support Norwalk virus replication (15). Contrasting this finding, a more recent study reported a change in viral genome from 24 to 48 hpi in naïve macrophages infected with the pandemic GII.4 strain of HuNoV but the lack of using inactivated virus as a control makes these data difficult to interpret (16). This is important because stool inoculum contains factors that may contribute to epithelial responses. Using ileal HIEs cocultured with naïve macrophages, this study, however, reported no significant change in HuNoV replication, which aligns with our current findings. Analysis of human intestinal biopsies from infected persons has not indicated such dual tropism *in vivo* thus far (12, 14). However, other reports using *in vivo* models such as zebrafish and piglets challenged with HuNoV demonstrated the presence of HuNoV viral capsid antigen, double-stranded RNA, or both in intestinal macrophages and other immune cells (29, 30). Roux et al. (29) analyzed specific macrophage cell clusters with high viral reads in zebrafish and showed high expression of genes associated with phagocytosis and pro-inflammatory M1 markers in the absence of detecting enterocyte transcripts. Again, these data remain difficult to interpret because inactivated virus was not used as a control. These findings suggest that certain macrophage subtypes may support HuNoV replication beyond their phagocytosis capacity. However, whether infectious viral progeny is produced from these identified macrophage subtypes remains undetermined.

Third, we showed that both naïve and activated macrophages were able to sense and phagocytose HuNoV-infected epithelial cells with an increase in phagocytic capability elicited by pro-inflammatory M1 macrophages. The pathogen sensing and phagocytic activity by macrophages has also been demonstrated in pathogenic *E. coli*. Noel et al. showed that apically infected *E.coli* was detected and phagocytosed by macrophages localized on the opposite side of the epithelial monolayer following an overnight infection (31). In line with this, our data showed an engulfment of HuNoV VP1-positive epithelial cells by both naïve and activated macrophages following an overnight infection, and this further supports our hypothesis that macrophages play a role in actively phagocytosing virus-infected cells (14). While our data do not exclude the possibility of macrophages becoming infected by HuNoV at later stages, future studies assessing infection in naïve and activated macrophages are needed to clarify this potential outcome.

In addition to the conventional role of pathogen recognition and clearance, we also showed a synergistic effect in cytokine production by macrophages when cocultured with HIEs in the absence of HuNoV infection. While it is unknown whether the cytokines are produced from the macrophages or HIEs, apical secretion of IL-6, IP-10, MIG, MIP-1α, MIP-1β, and RANTES was enhanced by macrophages. We also observed a robust induction of Th1/2 cytokines and growth factors secreted in the basolateral compartments, and this includes IP-10, MIG, MCP-3, MIP-1α, MIP-1β, RANTES, IL-2, IFN-γ, TNF-α, IL12p40, GM-CSF, IL-1α, IL-6, and PDGF-AB. A majority of these basolaterally secreted mediators exert pro-inflammatory functions aimed at promoting leukocyte recruitment, enhancing antigen presentation and driving the differentiation and activation of immune cells mobilized to the inflammatory site (32, 33). Our coculture systems that evaluated different subtypes of macrophages indicate some cytokines are made by specific subtypes, which extend previous results reporting IL-6, IL-8, and IFN-γ as the main cytokines secreted by HIE-M0 macrophage cocultures (31). Additionally, anti-inflammatory mediators IL-10 and IL-1RA were simultaneously induced. These results highlight key secretory features at the epithelial-macrophage interface whereby opposing signals are induced, and these may be critical in inducing pro-inflammatory functions as well as restoring immune homeostasis and circumventing excessive inflammation.

Contrary to the cytokine response in epithelial-macrophage crosstalk, responses following HuNoV infection in HIEs and HIE-macrophage cocultures were comparatively reduced in magnitude, and donor-to-donor difference was noticeably more profound. Nonetheless, we observed increased expression of chemokines (IP-10, MIP-1α, and RANTES) and acute-phase inflammatory mediators (IL-1α, IL1-RA, IL-6, TNF-α, and TNF-β) in the apical compartments of infected HIEs cocultured with activated macrophages. This induction of cytokine responses appears to be specific to HIEs cocultured with activated macrophages as greater than twofold increases in cytokine expression were not observed in infected HIEs and HIEs cocultured with naïve macrophages. This observation highlights the potential requirement of macrophage activation for the cytokine induction at an early time period during HuNoV replication. Previous studies primarily focused on the systemic immune responses to HuNoV infection. Sera collected from one human volunteer challenge study reported an elevation in Th1 and Th2 cytokines and chemokines including IFN-γ, IL-6, IL-8, IL-12p70, MCP, and TNF-α as an acute response to HuNoV infection (5, 6). A longitudinal study of elderly hospitalized patients also indicated a Th1-type response with an elevation of CXCL-9 and IP-10, macrophage migration inhibitory factor (MIF), IL-18, and secreted IL-2 receptor α in HuNoV-infected patients compared to controls (7). Yet these do not fully represent the mucosal responses during the early stages of infection. The increase in IP-10 expression observed in our study is particularly noteworthy, as its upregulation following HuNoV inoculation has been consistently documented in multiple studies at both transcriptional and protein levels (34–36). The relationship of fold increases in both the gene and protein expression of IP-10 with HuNoV replication was explored by Chan et al. (34). In this study, IP-10 and IFI44L emerged as the two most upregulated genes following HuNoV inoculation in HIEs, with their upregulation independently correlating with an increase in cell-associated HuNoV RNA. While IP-10 secretion was detected at 72 hpi, HuNoV-induced IP-10 secretion positively correlated with supernatant and cell-associated viral RNA increase. However, again, a notable limitation of this study is the lack of inactivated virus as a control. Overall, our findings, in conjunction with previous reports, indicate that HuNoV replication drives IP-10 transcription and subsequent protein secretion. Specifically, in HIEs cocultured with activated macrophages, IP-10 production was detectable as early as 24 hpi, highlighting its role in the early host immune response to HuNoV infection. Minimal cytokine secretion was observed in the basolateral compartments in both HIEs and HIE-macrophage cocultures, likely due to the need for multiple rounds of viral replication to adequately stimulate cytokine production toward the basement membrane.

In conclusion, our study explored the potential outcome of both naïve and activated macrophages in influencing the intestinal epithelium and identified secretory mediators unique to the epithelial-macrophage environment in the absence of enteric infection. Furthermore, we provide evidence that a subtype of activated macrophage is more proficient in responding to and phagocytizing HuNoV-infected cells. In addition, we defined the early cytokine response to HuNoV-infected HIEs that may help understanding factors initiating an epithelial response toward HuNoV. The human macrophage-enteroid coculture models described here provide an opportunity to examine macrophage activation in pathogen recognition and explore targetable host factors unique to epithelial-macrophage crosstalk. This model is valuable for studying the pathophysiology of other enteric pathogens, such as *Shigella* and *Salmonella*, where macrophages play a key role in dissemination. Additionally, it serves as an alternative platform for testing antimicrobial agents or immunomodulators.

## MATERIALS AND METHODS

### Monocyte isolation and differentiation of macrophages

Human monocytes were isolated from buffy coats obtained from healthy blood donors by a two-step gradient centrifugation followed by an additional step using Human Pan Monocyte Isolation kit (Miltenyi Biotec). Differentiation of macrophage subtypes was achieved as described previously (26, 37). Briefly, isolated monocytes were cultured at a density of 1 × 10^5^ cells/cm^2^ using Cellstar cell culture plates with cell repellent surfaces (Greiner Bio-one) cultured in macrophage growth medium to induce macrophage differentiation was induced with medium change every other day. The macrophage growth medium consists of RPMI-1640 medium ATCC modification (ThermoFisher) supplemented with 10% heat-inactivated fetal bovine serum (Sigma), 1× MEM non-essential amino acids (Sigma), 1 mM sodium pyruvate (Sigma), 55 µM β-mercaptoethanol (Gibco), and 100 ng/mL M-CSF (PeproTech). After 5-days differentiation, naïve M0 macrophages were stimulated with either 100 ng/mL LPS (Sigma) and 20 ng/mL IFN-γ (PeproTech) or 50 ng/mL IL-4 (PeproTech) in the macrophage growth medium for 48 h to generate pro-inflammatory M1 and anti-inflammatory M2 macrophages. For M0 macrophages, no cytokine was added to the macrophage growth medium and continued for culture until 7-days differentiation.

### HIEs and viruses

Jejunal (J2) HIE cultures were from an HIE bank maintained by the Gastrointestinal Experimental Model Systems (GEMS) Core of the Texas Medical Center Digestive Diseases Center (TMC DDC). HIE cultures were generated from surgical specimens acquired from bariatric surgery and propagated as multilobular, 3-dimensional (3D) structures embedded in Matrigel as previously described (10, 19, 20). For differentiating monolayers on Transwell inserts, 3D jejunal HIEs were dissociated to single cell suspension by trypsinization and seeded on human collagen type IV (33 µg/mL; Sigma)-coated Transwell inserts (0.33 cm^2^, 3 µm pore size, polyester membrane; Corning) at a cell density of 2.5 × 10^5^ cells per well. Jejunal HIE monolayers were first cultured in proliferation IntestiCult Organoid Growth Medium (OGMp), which contained 10 µM ROCK inhibitor Y-27632 for 1 day. After 1 day of growth, the HIE monolayers were differentiated using the differentiation OGM (OGMd) for 5 days. Details on the compositions of media used for HIE maintenance in 3D cultures and commercial Intesticult OGM media have been described previously (20). Stool filtrate containing HuNoV GII.4[P31] (Sydney 2012 variant) was prepared as described previously (10). Briefly, 20 mL sterile 0.01 M phosphate-buffered saline (PBS) was added to 20 g of HuNoV-positive stool, homogenized by vortexing and sonication three times for 1 min. The sonicated suspension was centrifuged at 1,500 × *g* for 10 min at 4°C. The supernatant was transferred to a fresh tube, and centrifugation was repeated. The resultant supernatant was passed serially through 5, 1.2, 0.8, 0.45, and 0.22 µm filters to remove bacterial contaminants. The filtered product was frozen in aliquots at −80°C until used. Preparation of γ-irradiated HuNoV stool filtrate has been described previously (9).

### Generation of HIE-macrophage cocultures

HIE-macrophage cocultures were generated by following a previously published protocol with some modifications (25). In brief, differentiated HIE monolayers on Transwell inserts were inverted and placed inside empty 12-well plates. Macrophages (10^5^ naïve macrophages or activated macrophages) were added to the top surfaces of the inverted Transwell inserts. Cells were attached for 2 h at 37°C in 5% CO_2_. Next, the inserts were returned to their original orientations with HIEs on the top and macrophages at the bottom and returned to 24-well plates. Fresh OGMd medium was replaced in the apical compartments of the HIE-macrophage cocultures and OGMd medium supplemented with M-CSF (20 ng/mL, PeproTech) was added into the basolateral compartments.

### HuNoV infection in HIEs and HIE-macrophage cocultures

HIE monolayers were washed apically once with CMGF^−^ medium and inoculated with HuNoV stool filtrate containing 4.2 × 10^5^ GE, γ-irradiated HuNoV stool filtrate diluted in CMGF^−^ medium supplemented with 500 µM bile acid (GCDCA) or CMGF^−^ medium only for 2 h at 37°C. The apical compartments were washed three times with CMGF^−^ medium to remove unbound virus, and fresh OGMd medium supplemented with 500 µm GCDCA was added. OGMd medium supplemented with 20 ng/mL of M-CSF was added to the basolateral compartments of HIEs and HIEs cocultured with macrophages.

### Quantification of viral replication by RT-qPCR

RNA extraction and RT-qPCR have been described previously (20). Briefly, total RNA was extracted from 100 µL of supernatants collected from both the apical and basolateral compartments, as well as the membranes containing HIE monolayers or with macrophages using the KingFisher Flex purification system and MagMAX-96 viral RNA isolation kit. RNA extracted at 2 hpi was used as a baseline to determine the level of cell-associated virus. The primer pair and probe COG2R/QNIF2d/QNIFS were used for the detection of GII genotypes (38). RT-qPCR was performed using qScript XLT One-Step RT-qPCR ToughMix reagent with ROX reference dye (Quanta Biosciences). Reactions were performed on an Applied Biosystems StepOnePlus thermocycler using the following conditions: 50°C (15 min), 95°C (5 min) followed by 40 cycles of 95°C (15 s) and 60°C (35 s). A standard curve based on a recombinant HuNoV RNA transcript was used to quantitate viral GE in RNA samples. The limit of detection was 20 GEs. A threshold for successful viral replication was established by considering a 0.5 increase in log_10_(GE) after 24 hpi relative to the genomic RNA at 2 hpi as described previously (19).

### H&E and immunofluorescence staining of HIEs and HIE-macrophage cocultures

For visualization of adherent macrophages, sectioning of paraformaldehyde (PFA)-fixed HIEs and HIE-macrophage cocultures was prepared as described previously (14). To assess macrophage phagocytosis following HuNoV infection, Transwells containing mock-treated, γ-irradiated HuNoV and HuNoV-infected HIEs and HIE-macrophage cocultures were fixed with 4% PFA and permeabilized with 0.2% Triton X-100 for 30 and 15 min at room temperature, respectively. Cells were blocked for 2 h in 5% IgG-free bovine serum album, 1% horse serum, and 1% goat serum diluted in PBS for 1 h at room temperature. Primary antibodies including guinea pig anti-GII.4/2012 virus-like particles at a 1:1,000 dilution (Covance, Princeton, NJ) (39), fluorescence-conjugated antisera to human CK8 (563614, BD) (1:100), and CD68 (ab955, Abcam) (1:100) were added to both compartments and incubated overnight at 4°C. After PBS washes, Alexa Fluor 488-conjugated anti-guinea pig secondary antibody (1:2,000, Invitrogen) was applied and nuclei were stained with 4,6-diamidino-2-phenylindole (DAPI) (300 nM) for 5 min at room temperature. Insert membranes containing monolayers were excised and mounted in prolong gold (ThermoFisher).

### Confocal microscopy

Confocal images were captured using either a ZEISS LSM 980 confocal microscope with Airyscan 2 using ZEISS ZEN 3.5 (blue edition) software or a Nikon A1-Rs confocal laser scanning microscope using NIS-Elements Viewer 4.20 with 20× and 40× oil objectives. Images were prepared using Imaris software (Version 8.4.2, Bitplane, Switzerland). For quantitative analysis of HuNoV-infected cells, the entire HIE monolayer was inspected to identify VP1 positive cells, and FOV was defined by VP1 positivity. Numbers of CK8-positive, CD68-positive or CK8-, CD68 double-positive cells containing VP1 were quantified and normalized to the number of nuclei within the FOV. The phagocytosis index was determined by the number of CD68/CK8/VP1 triple-positive cells and divided by the total number of nuclei in the FOV. VP1 positivity was also inspected in macrophages attached on the basolateral side of Transwell inserts.

### Flow cytometry

The following human-specific monoclonal antibodies were used to identify macrophages: MϕP9 (anti-CD14, BUV395-conjugated), Y1/82A (anti-CD68, PE-CF594-conjugated), B7-1 (anti-CD80, Alexa Fluor 647-conjugated) and 19.2 (anti-CD206, FITC-conjugated) (BD Biosciences). Cells were blocked in BD Fc Block (BD Biosciences) for 15 min at 4°C and incubated with antibodies for 30 min at 4°C. Cells were washed three times and resuspended in staining buffer before analysis and analyzed using a BD LSR Fortessa and FACSDIVA software (BD Biosciences). Data were analyzed using FlowJo software (v10.1).

### Cytokine analysis

Supernatant collected from the apical and basolateral compartments of the HIE and HIE-macrophage cultures (mock, γ-irradiated, and HuNoV-infected) was collected at 24 h post-infection and stored at −80°C for further analysis. Milliplex Human Cytokine/Chemokine/Growth Factor Panel A (Millipore EMD) was used to determine the cytokine levels according to the manufacturer’s protocol. All results were interpolated using data from the standards included in the kit.

### Statistical analysis

All experiments were performed with at least three independent PBMC donors. All statistical analyses were performed using Graphpad Prism version 10.2.3 for Macintosh (Graphpad Software, La Jolla, CA, USA). *P* values of < 0.05 were considered statistically significant. The sparse Partial Least Squares Discriminant Analysis (sPLSDA) method implemented in the R package mixOmics (40) was used to distinguish HIEs based on exposure to macrophage subtypes. Samples were plotted as scatterplots using the top two sPLSDA components; loadings on the first sPLSDA component were plotted for most informative cytokines.

## SUPPLEMENTAL MATERIAL

Fig. S1 mbio.01180-25-s0001.pdf Cytokine stimulation induces differentiation of macrophage subtypes using human peripheral blood-derived monocytes.

Fig. S2 mbio.01180-25-s0002.pdf Increased CD80 and CD206 in pro-inflammatory M1 and anti-inflammatory M2 macrophages.

Fig. S3 mbio.01180-25-s0003.pdf Barrier integrity remains unchanged upon macrophage integration to differentiated HIEs.

Fig. S4 mbio.01180-25-s0004.pdf The presence of macrophage subtypes does not alter barrier function in OGM-cultured HIE.

Fig. S5 mbio.01180-25-s0005.pdf HuNoV replication does not alter the barrier integrity of differentiated HIEs.

Fig. S6 mbio.01180-25-s0006.pdf IP-10 and MIG are preferentially secreted apically in all HIE-macrophage cocultures.

Fig. S7 mbio.01180-25-s0007.pdf Addition of macrophages induces basolateral secretion of chemokines and Th1/Th2 cytokines.

Fig. S8 mbio.01180-25-s0008.pdf Group separation by basolateral cytokine response revealed distinct separation of HIE-macrophage cocultures.

ASM does not own the copyrights to Supplemental Material that may be linked to, or accessed through, an article. The authors have granted ASM a non-exclusive, world-wide license to publish the Supplemental Material files. Please contact the corresponding author directly for reuse.
