# Supplementary material for: Macrophage phagocytosis of human norovirus-infected cells in an ex vivo human enteroid-macrophage coculture model
Source: mBio. 2025 Jul 9;16(8):e01180-25. doi: 10.1128/mbio.01180-25 (PMC12345152; doi:10.1128/mbio.01180-25)
Supplement: Fig. S1 — Cytokine stimulation induces differentiation of macrophage subtypes using human peripheral blood-derived monocytes. [file mbio.01180-25-s0001.pdf]

A.

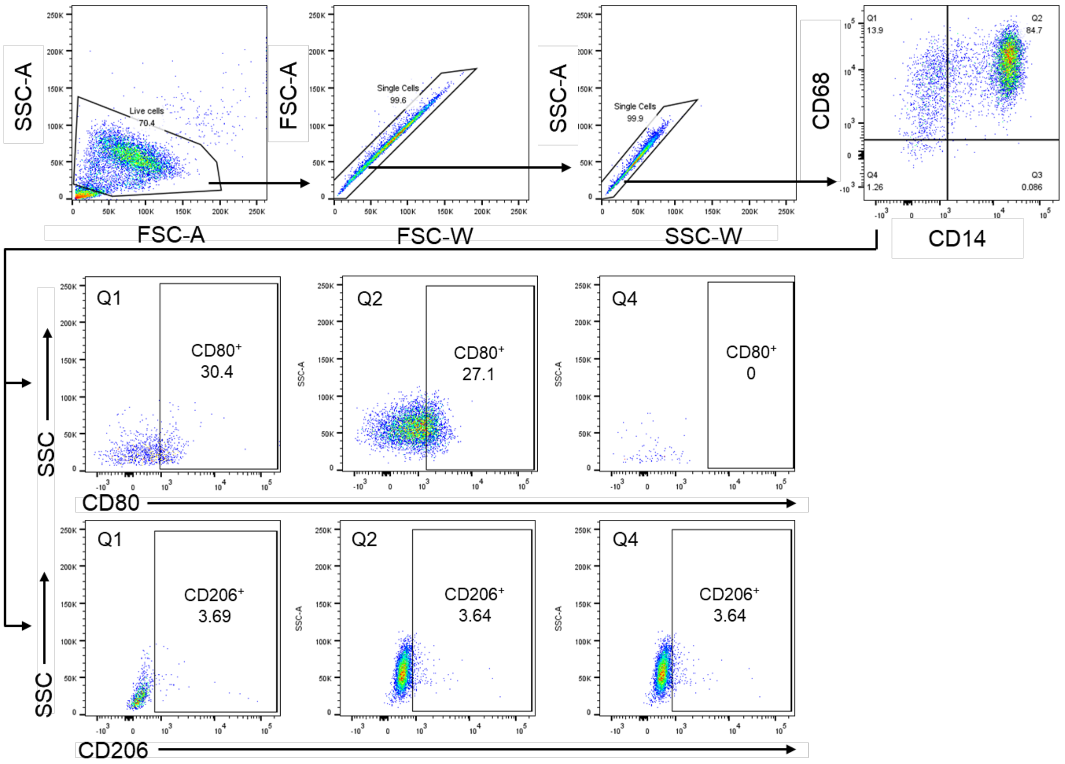

SSC-A
FSC-A
Live cells
70.4
FSC-A
FSC-W
Single Cells
99.6
SSC-A
SSC-W
Single Cells
99.9
CD68
CD14
Q1
13.9
Q2
84.7
Q4
1.26
Q3
0.086
Q1
CD80+
30.4
Q2
CD80+
27.1
Q4
CD80+
0
SSC
CD80
Q1
CD206+
3.69
Q2
CD206+
3.64
Q4
CD206+
3.64
SSC
CD206

B.

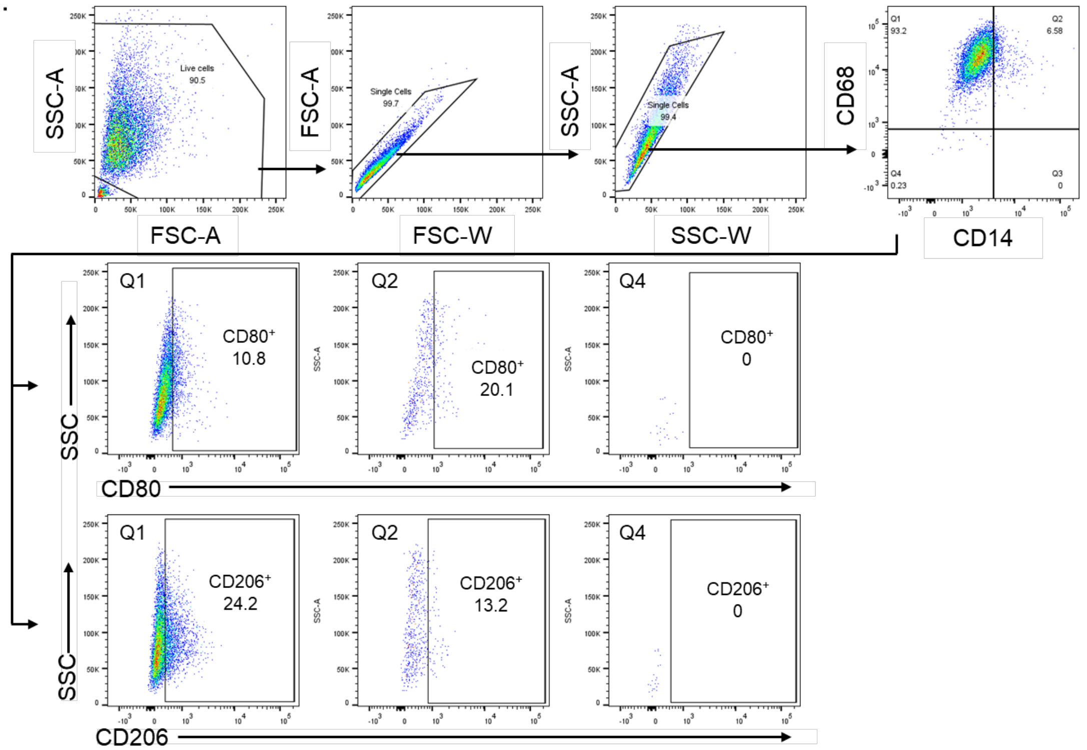

SSC-A
FSC-A
Live cells
90.5
FSC-A
FSC-W
Single Cells
99.7
SSC-A
SSC-W
Single Cells
99.4
CD68
CD14
Q1
93.2
Q2
6.58
Q4
0.23
Q3
0
Q1
CD80+
10.8
Q2
CD80+
20.1
Q4
CD80+
0
SSC
CD80
Q1
CD206+
24.2
Q2
CD206+
13.2
Q4
CD206+
0
SSC
CD206

C.

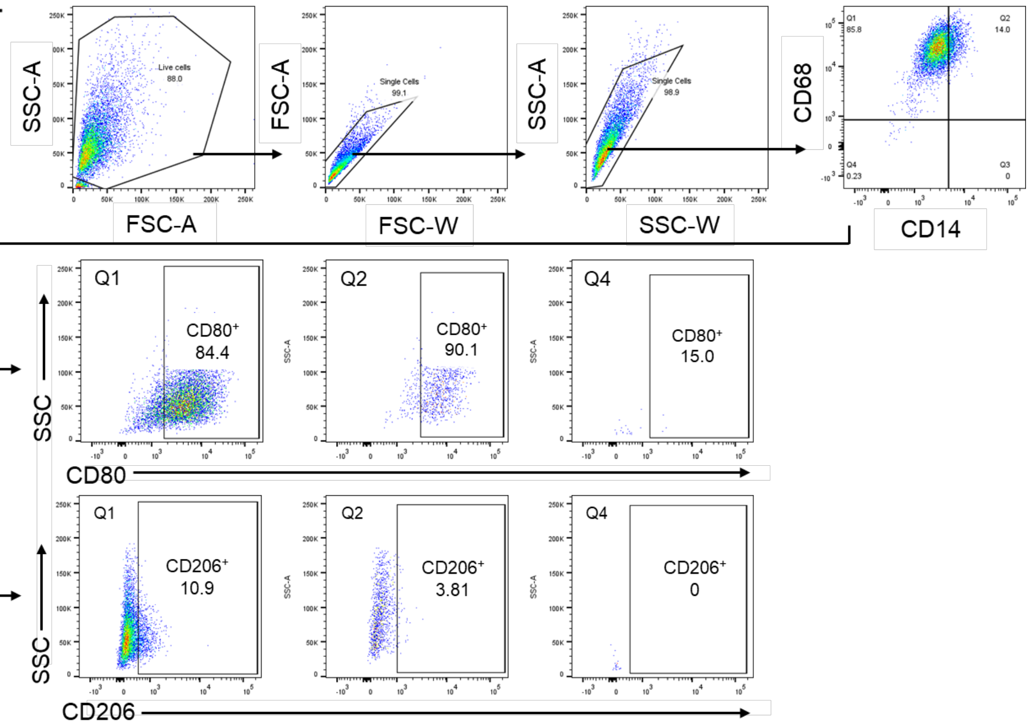
SSC-A
FSC-A
Live cells
88.0
FSC-A
FSC-W
Single Cells
99.1
SSC-A
SSC-W
Single Cells
98.9
CD68
CD14
Q1
85.8
Q2
14.0
Q4
0.23
Q3
0
Q1
CD80+
84.4
Q2
CD80+
90.1
Q4
CD80+
15.0
SSC
CD80
Q1
CD206+
10.9
Q2
CD206+
3.81
Q4
CD206+
0
SSC
CD206

D.

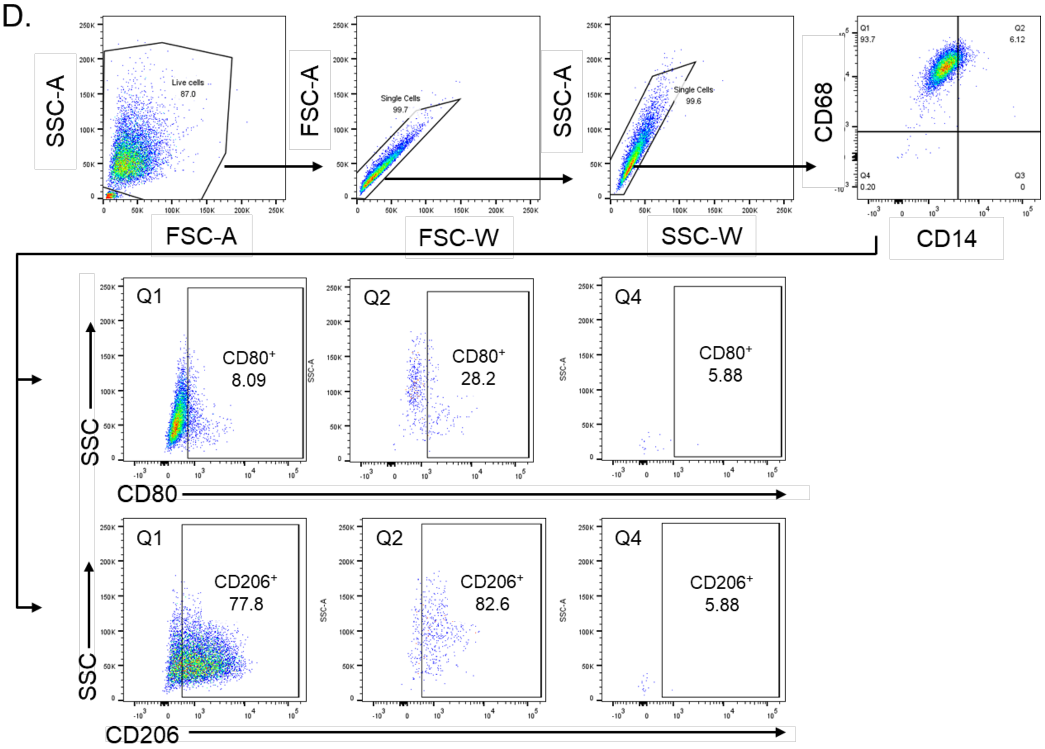
SSC-A
FSC-A
Live cells
87.0
FSC-A
FSC-W
Single Cells
99.7
SSC-A
SSC-W
Single Cells
99.6
CD68
CD14
Q1
93.7
Q2
6.12
Q4
0.20
Q3
0
Q1
CD80+
8.09
Q2
CD80+
28.2
Q4
CD80+
5.88
SSC
CD80
Q1
CD206+
77.8
Q2
CD206+
82.6
Q4
CD206+
5.88
SSC
CD206

FIG. S1 Cytokine stimulation induces differentiation of macrophage subtypes using human peripheral blood-derived monocytes. Representative flow cytometry plots showing the gating strategy to assess surface expression of CD14, CD68, CD80 and CD206 on **A.** monocyte, **B.** naïve M0, **C.** pro-inflammatory M1 and **D.** anti-inflammatory M2 macrophages. Data are representative of five independent donors.
