# Supplementary material for: Macrophage phagocytosis of human norovirus-infected cells in an ex vivo human enteroid-macrophage coculture model
Source: mBio. 2025 Jul 9;16(8):e01180-25. doi: 10.1128/mbio.01180-25 (PMC12345152; doi:10.1128/mbio.01180-25)
Supplement: Fig. S2 — Increased CD80 and CD206 in pro-inflammatory M1 and anti-inflammatory M2 macrophages. [file mbio.01180-25-s0002.pdf]

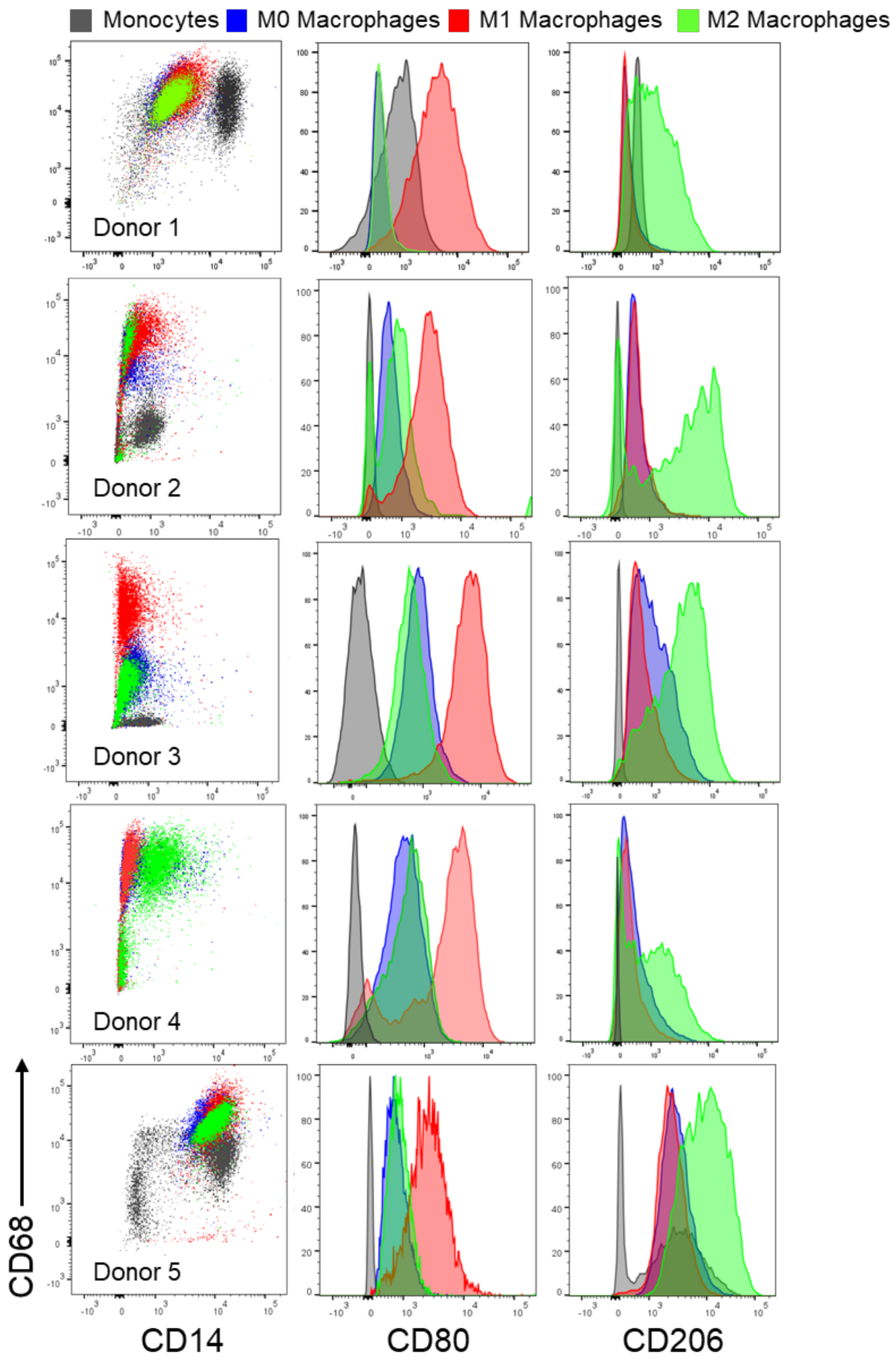
Monocytes
M0 Macrophages
M1 Macrophages
M2 Macrophages
Donor 1
Donor 2
Donor 3
Donor 4
Donor 5
CD68
CD14
CD80
CD206

FIG. S2 Increased CD80 and CD206 in pro-inflammatory M1 and anti-inflammatory M2 macrophages. The expression of CD14, CD68, CD80 and CD206 on isolated monocytes (grey), naïve M0 (blue), pro-inflammatory M1 (red) and anti-inflammatory M2 (green) macrophages were assessed using flow cytometry. The dot plots and histograms, illustrating macrophage heterogeneity, were superimposed to visualize data from five PBMC donors, with each panel displaying data from a single donor.
