# Supplementary material for: Macrophage phagocytosis of human norovirus-infected cells in an ex vivo human enteroid-macrophage coculture model
Source: mBio. 2025 Jul 9;16(8):e01180-25. doi: 10.1128/mbio.01180-25 (PMC12345152; doi:10.1128/mbio.01180-25)
Supplement: Fig. S3 — Barrier integrity remains unchanged upon macrophage integration to differentiated HIEs. [file mbio.01180-25-s0003.pdf]

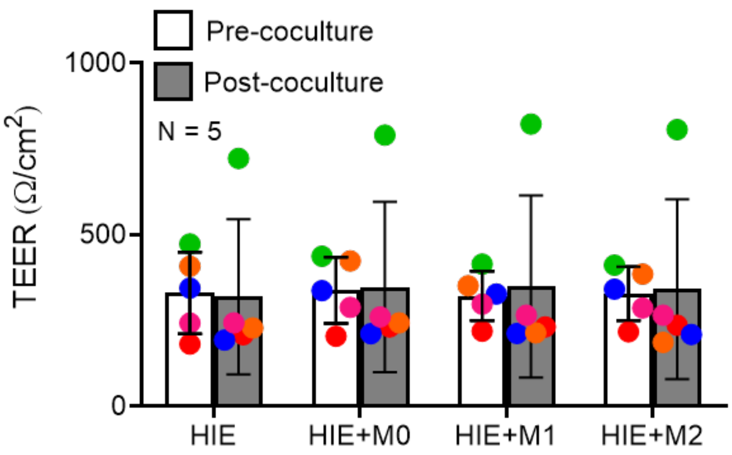

FIG. S3 Barrier integrity remains unchanged upon macrophage integration to differentiated HIEs. HIEs were differentiated in Intesticult™ OGM media for five days and subjected to coculture with naïve M0 or activated macrophages (pro-inflammatory M1 or anti-inflammatory M2 macrophages). Transepithelial resistance (TEER) was measured pre-coculture (white bars) and at 24-hour post coculture (grey bars) in differentiated HIEs and HIE-macrophage cocultures. Data are represented as mean ± SD and compiled from five experiments. Each dot represents monocyte-derived macrophages from a single donor's PBMCs used for one coculture experiment. TEER values shown here were used for the analysis shown in **Fig. 1E**.
