# Supplementary material for: Macrophage phagocytosis of human norovirus-infected cells in an ex vivo human enteroid-macrophage coculture model
Source: mBio. 2025 Jul 9;16(8):e01180-25. doi: 10.1128/mbio.01180-25 (PMC12345152; doi:10.1128/mbio.01180-25)
Supplement: Fig. S4 — The presence of macrophage subtypes does not alter barrier function in OGM-cultured HIE. [file mbio.01180-25-s0004.pdf]

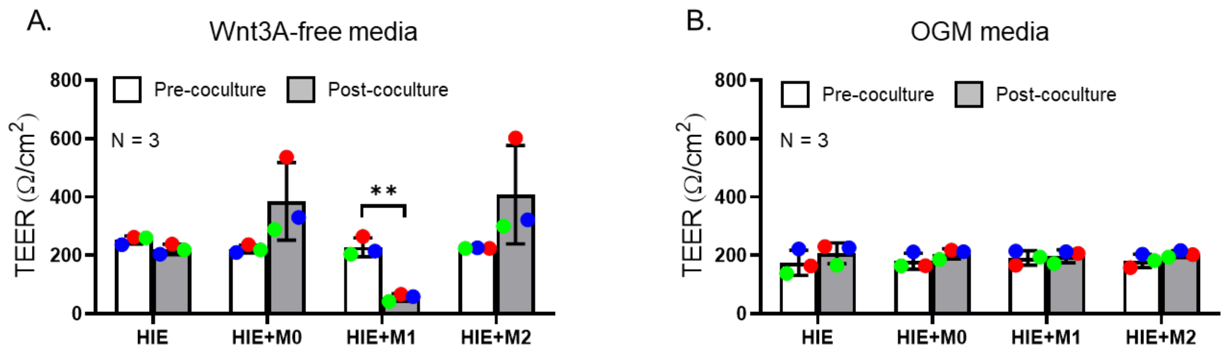

FIG. S4 The presence of macrophage subtypes does not alter barrier function in OGM-cultured HIE. HIEs were differentiated in **A.** Wnt3A-free differentiation media or **B.** Intesticult™ OGM media for five days and subjected to coculture with naïve (M0 macrophages) or activated macrophages (M1 or M2 macrophages). Transepithelial resistance (TEER) was measured pre-coculture (white bars) and at 1-day post coculture (grey bars) conditions in differentiated HIE and HIE-macrophage cocultures. Data are represented as mean ± SD and compiled from three experiments. Each dot represents monocyte-derived macrophages from a single donor's PBMCs used for one coculture experiment. Statistical significance was determined using Students' t-test; $^{**}p < 0.01$.
