# Supplementary material for: Macrophage phagocytosis of human norovirus-infected cells in an ex vivo human enteroid-macrophage coculture model
Source: mBio. 2025 Jul 9;16(8):e01180-25. doi: 10.1128/mbio.01180-25 (PMC12345152; doi:10.1128/mbio.01180-25)
Supplement: Fig. S5 — HuNoV replication does not alter the barrier integrity of differentiated HIEs. [file mbio.01180-25-s0005.pdf]

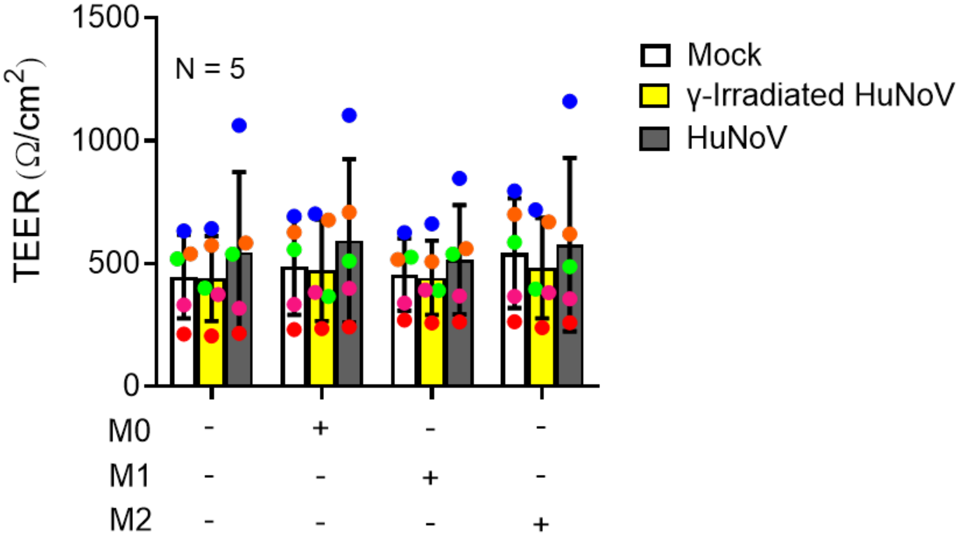

FIG. S5 HuNoV replication does not alter the barrier integrity of differentiated HIEs. Measurement of transepithelial resistance (TEER) of mock-treated (white bar), γ-irradiated HuNoV-infected (yellow) and HuNoV-infected (grey) HIE and HIE-macrophage cocultures at 24h post infection. Data are represented as mean ± SD and compiled from five experiments. Each color corresponds to monocyte-derived macrophages derived from the PBMCs of a single donor used for one coculture experiment.
