# Supplementary material for: Macrophage phagocytosis of human norovirus-infected cells in an ex vivo human enteroid-macrophage coculture model
Source: mBio. 2025 Jul 9;16(8):e01180-25. doi: 10.1128/mbio.01180-25 (PMC12345152; doi:10.1128/mbio.01180-25)
Supplement: Fig. S6 — IP-10 and MIG are preferentially secreted apically in all HIE-macrophage cocultures. [file mbio.01180-25-s0006.pdf]

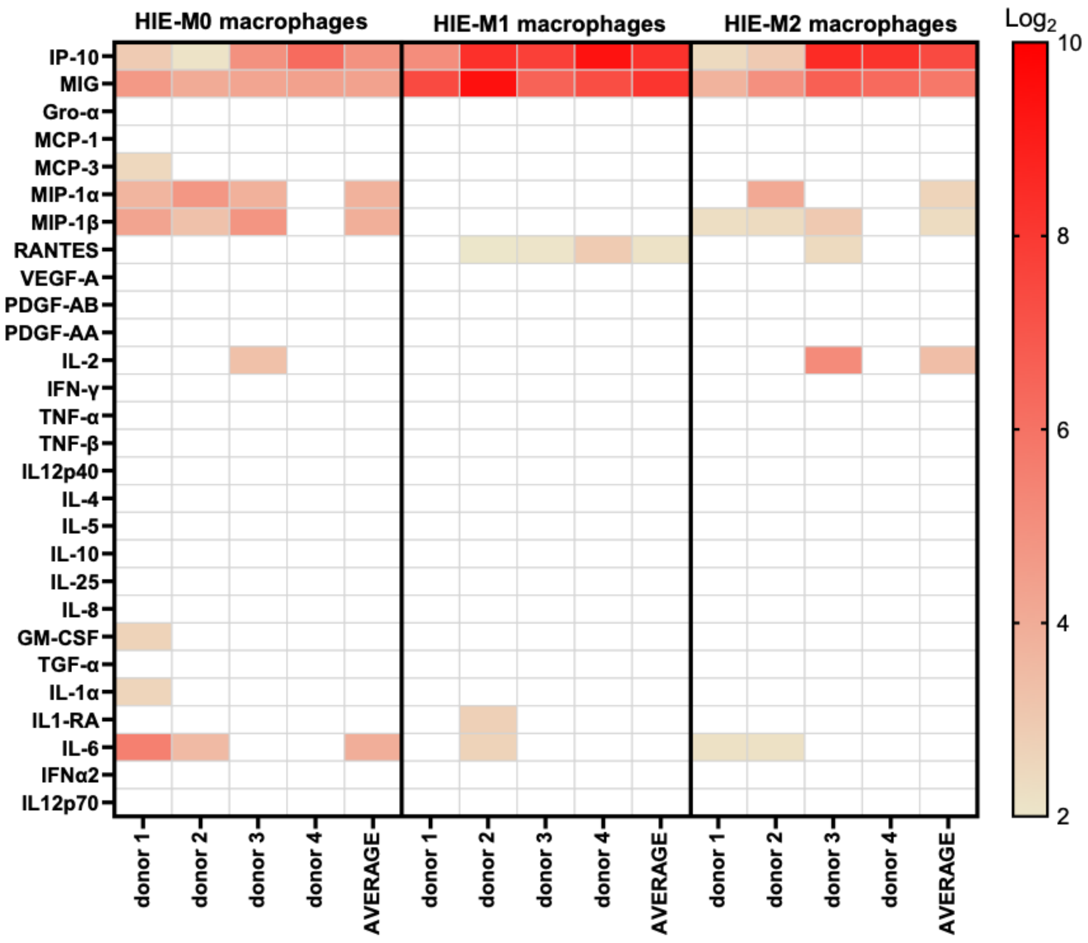

FIG. S6 IP-10 and MIG are preferentially secreted apically in all HIE-macrophage cocultures. Supernatants collected from HIE-macrophage cocultures were assessed for cytokine secretion. The panel shows the $\log_2$ fold increases in cytokine levels relative to the levels measured in HIE alone. Each row represents an individual cytokine assessed in the multiplex assay while each column indicates an individual PBMC donor and the average value across all donors. Cytokines with less than a 2-fold change over HIE alone are depicted as white.
