# Supplementary material for: Macrophage phagocytosis of human norovirus-infected cells in an ex vivo human enteroid-macrophage coculture model
Source: mBio. 2025 Jul 9;16(8):e01180-25. doi: 10.1128/mbio.01180-25 (PMC12345152; doi:10.1128/mbio.01180-25)
Supplement: Fig. S7 — Addition of macrophages induces basolateral secretion of chemokines and Th1/Th2 cytokines. [file mbio.01180-25-s0007.pdf]

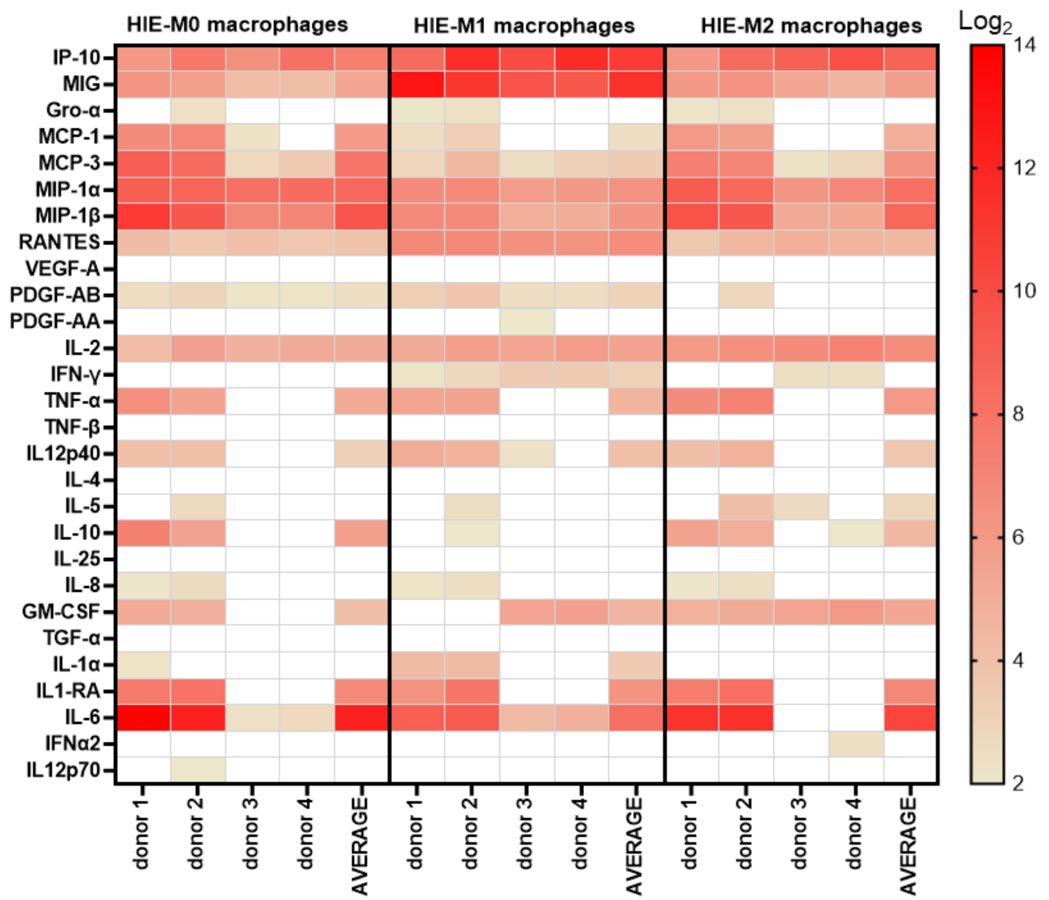

FIG. S7 Addition of macrophages induces basolateral secretion of chemokines and Th1/Th2 cytokines. Basolateral media collected from HIE-macrophage cocultures were assessed for cytokine secretion. The panel shows the log_2 fold increases in cytokine levels relative to the levels measured in HIE alone. Each row represents an individual cytokine assessed in the multiplex assay while each column indicates an individual PBMC donor and the average value across all donors. Cytokines with less than a 2-fold change over HIE alone are depicted as white.
