# Supplementary material for: Macrophage phagocytosis of human norovirus-infected cells in an ex vivo human enteroid-macrophage coculture model
Source: mBio. 2025 Jul 9;16(8):e01180-25. doi: 10.1128/mbio.01180-25 (PMC12345152; doi:10.1128/mbio.01180-25)
Supplement: Fig. S8 — Group separation by basolateral cytokine response revealed distinct separation of HIE-macrophage cocultures. [file mbio.01180-25-s0008.pdf]

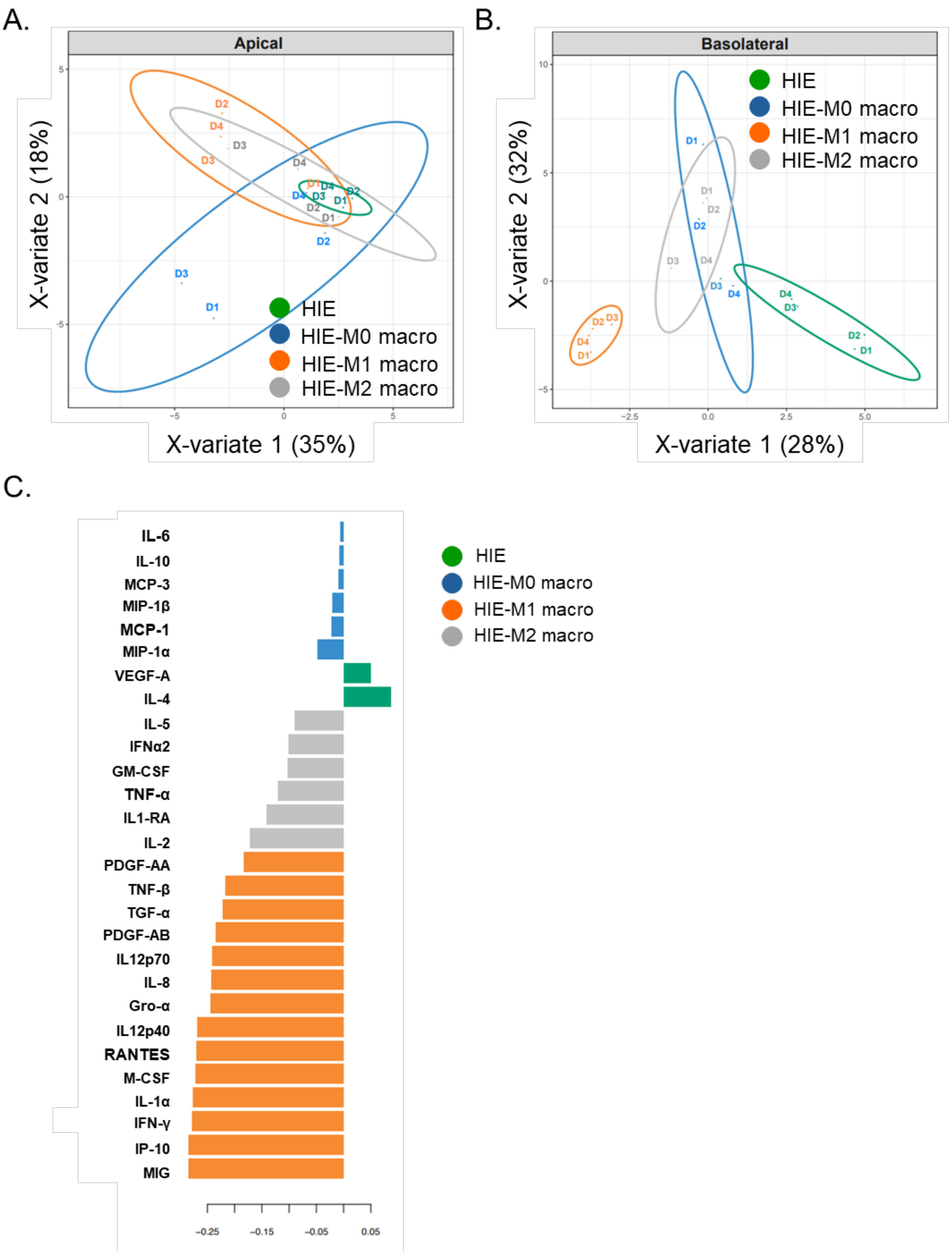
A.
Apical
X-variate 2 (18%)
X-variate 1 (35%)
HIE
HIE-M0 macro
HIE-M1 macro
HIE-M2 macro
B.
Basolateral
X-variate 2 (32%)
X-variate 1 (28%)
HIE
HIE-M0 macro
HIE-M1 macro
HIE-M2 macro
C.
IL-6
IL-10
MCP-3
MIP-1β
MCP-1
MIP-1α
VEGF-A
IL-4
IL-5
IFNα2
GM-CSF
TNF-α
IL1-RA
IL-2
PDGF-AA
TNF-β
TGF-α
PDGF-AB
IL12p70
IL-8
Gro-α
IL12p40
RANTES
M-CSF
IL-1α
IFN-γ
IP-10
MIG
−0.25
−0.15
−0.05
0.05
HIE
HIE-M0 macro
HIE-M1 macro
HIE-M2 macro

FIG. S8 Group separation by basolateral cytokine response revealed distinct separation of HIE-macrophage cocultures. sPLSDA to differentiate between HIEs (green), HIE cocultured with M0 (blue), M1 (orange) or M2 macrophages (grey) based on cytokine response assessed in the **A.** apical and **B.** basolateral compartments. Each point represents one PBMC donor (denoted as D1, D2, D3 and D4), where isolated monocytes were used to establish an individual HIE-macrophage coculture experiment. **C.** Basolaterally-detected cytokines that contributed to X-variate 1 either to HIEs (green), HIE-M0 macrophage (blue), HIE-M1 macrophage (orange) or HIE-M2 macrophages (grey) clusters.
